# Characterization of a Polyethylene Glycol-Amphotericin B Conjugate Loaded with Free AMB for Improved Antifungal Efficacy

**DOI:** 10.1371/journal.pone.0152112

**Published:** 2016-03-23

**Authors:** Tessa Rui Min Tan, Kong Meng Hoi, Peiqing Zhang, Say Kong Ng

**Affiliations:** 1 Bioprocessing Technology Institute, Agency for Science, Technology and Research (A*STAR), Singapore, Singapore; 2 Department of Anatomy, Yong Loo Lin School of Medicine, National University of Singapore, Singapore, Singapore; 3 Department of Pharmacy, Faculty of Science, National University of Singapore, Singapore, Singapore; Tecnologico de Monterrey, MEXICO

## Abstract

Amphotericin B (AMB) is a highly hydrophobic antifungal, whose use is limited by its toxicity and poor solubility. To improve its solubility, AMB was reacted with a functionalized polyethylene glycol (PEG), yielding soluble complex AmB-PEG formulations that theoretically comprise of chemically conjugated AMB-PEG and free AMB that is physically associated with the conjugate. Reverse-phase chromatography and size exclusion chromatography methods using HPLC were developed to separate conjugated AMB-PEG and free AmB, enabling the further characterization of these formulations. Using HPLC and dynamic light scattering analyses, it was observed that the AMB-PEG 2 formulation, having a higher molar ratio of 2 AMB: 1 PEG, possesses more free AMB and has relatively larger particle diameters compared to the AMB-PEG 1 formulation, that consists of 1 AMB: 1 PEG. The identity of the conjugate was also verified using mass spectrometry. AMB-PEG 2 demonstrates improved antifungal efficacy relative to AMB-PEG 1, without a concurrent increase in *in vitro* toxicity to mammalian cells, implying that the additional loading of free AMB in the AMB-PEG formulation can potentially increase its therapeutic index. Compared to unconjugated AMB, AMB-PEG formulations are less toxic to mammalian cells *in vitro*, even though their MIC_50_ values are comparatively higher in a variety of fungal strains tested. Our *in vitro* results suggest that AMB-PEG 2 formulations are two times less toxic than unconjugated AMB with antifungal efficacy on *Candida albicans* and *Cryptococcus neoformans*.

## Introduction

Amphotericin B (AMB), a polyene antifungal agent, is frequently used to treat systemic fungal infections. Such infections are often opportunistic, and cause significant morbidity and mortality among immunocompromised individuals [1]. Due to its broad spectrum of activity, AMB remains the drug of choice for the treatment of many systemic fungal infections [2]. AMB exerts its antifungal effect via binding to and removing ergosterol from fungal cell membranes [3], via the formation of extramembranous aggregates [4]. While the binding affinity between AMB and cholesterol is lower, it is still toxic to mammalian cells [4]. Crystalline AMB is highly hydrophobic, virtually insoluble in water and only sparingly soluble in many organic solvents, making drug delivery challenging [5]. It has a low therapeutic index, and limitations of intravenous AMB therapy include severe side-effects [6] and poor aqueous solubility, which can potentially reduce its efficacy in certain fungal lesions [7]. The current conventional dosage form is a colloidal dispersion of AMB formulated with sodium deoxycholate (Fungizone). Nausea, fever and hypotension are manifestations of acute AMB deoxycholate toxicity, and are often infusion-related [8]. Organ damage, particularly to the kidneys, is drug-mediated and is a chronic side-effect of AMB treatment [9].

Various approaches have been employed to mitigate the adverse effects of AMB [10]. New dosage forms such as lipid formulations of AMB have been developed and are currently in use. Compared to Fungizone, liposomal or lipid complexes of AMB are less toxic, possess better aqueous solubility, and yet retain comparable *in vivo* antifungal efficacy [11]; however, as they can be prohibitively expensive, these formulations are not used extensively in many clinical settings [12]. Another method used is the chemical modification of the AMB molecule to enhance its activity and reduce non-specific binding by increasing its affinity to ergosterol relative to cholesterol, which could improve its therapeutic index [13]: AMB derivatives lacking the C2′-OH on the mycosamine arm bind to ergosterol but not to cholesterol, and have their antifungal efficacy maintained while being nontoxic to mammalian cells [14,15].

A third approach to enhance AMB’s aqueous solubility and reduce its toxicity is through the addition of a highly hydrophilic compound to AMB. One modification studied was the addition of arabinogalactan, a polysaccharide that possesses extraordinarily high water solubility, to AMB. The AMB-arabinogalactan conjugate displayed similar antifungal efficacies compared to the unconjugated AMB in certain fungal species, as well as reduced *in vivo* toxicity in mice [16]. Another modification that was considered was the addition of polyethylene glycol (PEG) to the AMB molecule. AMB-PEG conjugates have been synthesized by linking AMB to methoxy-PEG via carbamate linkages to its amine group, such that approximately half of the AMB is linked to PEG, with the rest being attached non-covalently. These compounds possess *in vitro* antifungal activity comparable to that of AMB deoxycholate, although their relative toxicity to mammalian cells was not investigated [17]. PEG has also been conjugated to AMB via its carboxylic acid group [18], generating less toxic compounds with demonstrated *in vivo* antifungal efficacy [19,20]. Other studies have used hydrolysable linkers to synthesize AMB-PEG conjugates that can act as prodrugs that generally demonstrate little *in vitro* antifungal activity. To effect antifungal activities, AMB was released *in vivo* via enzymatic1,6- benzyl elimination of the labile carbamate linkage between the two moieties [21], or through hydrolysis of pH-sensitive imine linkages at infection sites [22].

Since PEG is biologically inert, nontoxic and nonimmunogenic, it is widely used as a modifier for both protein and non-protein small drug molecules, and is thus particularly well-suited to function as a modifier for AMB. Covalent attachment of the highly hydrophilic PEG enhances the aqueous solubility of many compounds and can confer other desirable properties such as reduced immunogenicity, improved pharmacokinetic profiles and targeted biodistribution [23]. When conjugated to AMB, PEG improved the solubility and reduced the toxicity of the antifungal drug [21,22]. As such, AMB-PEG conjugates may be a promising candidate for future antifungal drug development.

To further investigate the use of AMB-PEG conjugates as antifungal therapeutics, we combined the concepts of AMB liposomal formulations with that of AMB-PEG conjugation. AMB-PEG mixtures with excess AMB were formulated and characterized with respect to their solubility, size and UV-visible absorption profiles. HPLC and MALDI-TOF were likewise performed to establish the elution profiles of the formulations and to subsequently verify the identity of the conjugate in the collected fractions. Finally, their antifungal efficacies and toxicities to mammalian cells were evaluated.

## Materials and Methods

### Preparation of AMB-PEG Formulations

AMB (Merck KGaA, Darmstadt, Germany) and MS(PEG)_4_ (MW: 333.3 Da, Thermo Fisher Scientific, Rockford, IL) were dissolved in anhydrous dimethylsulfoxide (DMSO) (Sigma-Aldrich, St. Louis, MO) to a concentration of 125 mM and 250 mM respectively. Different molar ratios of AMB to MS(PEG)_4_ were reacted in DMSO for 2 hours at room temperature, after which more DMSO was added to obtain an AMB concentration of 20 mM. The mixture was then dispersed in a phosphate buffered saline solution with 3 mM EDTA at pH 8 (PBS-EDTA) to a final concentration of 2 mM of AMB. To remove DMSO and unreacted MS(PEG)_4_ in the AMB-PEG formulation, concentration and diafiltration with PBS-EDTA was performed using Amicon Ultra 0.5 mL 10 kDa centrifugal filters (Millipore, Billerica, MA), which retained AMB-PEG, at 4°C. Dynamic Light Scattering (DLS) analysis (described below) was subsequently carried out on diafiltered and non-diafiltered samples, and it was established that the diafiltration step did not significantly affect the particle size distribution of the AMB-PEG formulation. AMB-PEG formulations were then filtered using a 0.22 μm PVDF syringe filter (Millipore) prior to *in vitro* toxicity and activity tests.

### UV-Visible Absorption Profile of AMB-PEG Formulations

20 mM of AMB-PEG formulations and unconjugated AMB in DMSO were dispersed in various solvents to a concentration of 2 mM. The mixtures were centrifuged at 14000 *g* for 10 minutes to remove particulate matter, and the supernatants collected for analysis. The UV absorption profiles of these supernatants were then obtained using the Nanodrop 2000 (Thermo Fisher Scientific). Buffers used for reconstitution were PBS-EDTA, and acetonitrile (ACN)-water-acetic acid mixtures at ratios of 20:75.7:4.3 and 48:47.7:4.3 v/v/v.

### Quantification of Diafiltered and 0.22 μm-Filtered AMB-PEG Formulations

As losses occur during the preparation of diafiltered and 0.22 μm-filtered AMB-PEG conjugate formulations, the concentrations of AMB in the formulations need to be quantified. Since the UV-visible absorption spectrum of all solutions of AMB-PEG in PBS-EDTA demonstrated three absorption maxima at 365, 385 and 409 nm, this property was used to quantify the concentrations of filtered AMB-PEG conjugate formulations. Unfiltered AMB-PEG samples were serially diluted in PBS-EDTA and their absorbance at the three aforementioned wavelengths acquired using a Nanodrop 2000. It was observed that at concentrations between 0.5 and 4 mM, the absorbance at 365 nm increases linearly with a corresponding increase in AMB-PEG concentration. As similarly diluted samples of filtered AMB-PEG formulations also had comparable gradients to that of the unfiltered formulation, a standard curve generated by the unfiltered formulation was thus used to quantify the concentrations of the sterile-filtered AMB-PEG. All concentrations of AMB-PEG were calculated and reported based on the concentration of AMB in the sample.

### Solubility and Stability of AMB-PEG Conjugate Formulations in Saline Solutions

AMB-PEG conjugate formulations in DMSO at AMB:PEG ratios of 1:1 and 2:1 were prepared at a concentration of 83.3 mM AMB. Increasing volumes of AMB-PEG was then added separately to tubes containing 500 μL of PBS-EDTA, over a volume range of 5 to 120 μL. The contents of each tube was mixed and centrifuged at 14000 *g* for 10 minutes. After centrifugation, supernatants were obtained from each sample, diluted appropriately and AMB-PEG concentrations quantified with Nanodrop based on their absorbance at 365 nm as described in 2.3 above. Supernatants obtained from each sample and samples that were diafiltered using a 10 kDa centrifugal filter were then stored at 4°C and after 4 weeks, centrifuged at 14000 *g* to observe for precipitation.

### AMB-PEG Particle Size Determination Using DLS

2 mM solutions of AMB-PEG were prepared in PBS-EDTA, and diafiltration carried out with PBS-EDTA using 10 kDa centrifugal filters (Millipore). The AMB-PEG retentate was adjusted to a concentration of 2 mM, and subsequently serially diluted to obtain solutions ranging in concentrations from 0.05 to 2 mM. The viscosities of the respective buffers used were obtained using a Vibro Viscometer SV-10 (A&D, Tokyo, Japan). These values and the temperature at which they were acquired were entered into the particle sizing software for analysis. Using a low-volume quartz cuvette (ZEN 2112) (Malvern Instruments, Worcestershire, UK), the relative scattering intensity of each 50 μL sample was measured using the Zetasizer Nano ZS (Malvern Instruments) at a fixed angle of 173°. A refractive index of 1.33 and dielectric constant of 0.1 was also used for particle size calculations at their respective controlled temperatures. The number of runs was determined automatically by the software, and each sample was analyzed thrice. Plots of relative scattering intensity against theoretical particle size, peak values, polydispersity indices and other parameters were acquired. Intensity-weighted particle diameters corresponding to the median and 90% size upper limit were also obtained and the error bars displayed denote the standard deviation of the mean of 3 independent measurements.

### HPLC Characterization of AMB-PEG Formulations

#### Reverse phase chromatography (RPC)

Samples were analyzed using a modified method adapted from Espada et al. [24], that was developed to quantify AMB in biological samples. Filtered AMB-PEG samples in PBS-EDTA were prepared as described above; unconjugated AMB was dissolved in DMSO, dispersed in 48% ACN, centrifuged for 10 minutes at 14000 *g* and passed through a 0.22 μm PVDF filter to remove any remaining particles. Unconjugated AMB in PBS-EDTA could not be used for this experiment due to its poor aqueous solubility. 50 μL of each AMB-PEG conjugate formulation and 10 μL of unconjugated AMB was applied separately into a BDS Hypersil C18 5 μm 250×4.6 mm column (Thermo Scientific) equilibrated with ACN-acetic acid-water (48:4.3:47.7 v/v/v) and eluted isocratically at a flow rate of 0.5 ml/min for 40 minutes. Eluted peaks were detected by a UV detector at 406 nm. Peak fractions were collected and analyzed using MALDI-TOF and size exclusion chromatography.

#### Size exclusion chromatography (SEC)

SEC was performed at a flow rate of 0.1 ml/min using a Superdex 75 PC 3.2/30 column (GE Healthcare, UK) equilibrated with the appropriate mobile phase. Eluted peaks were detected at 406 nm.

When a buffered 20% ACN mobile phase was used, 20 mM of AMB-PEG conjugate formulations and unconjugated AMB in DMSO were prepared and resuspended at 2 mM in ACN-acetic-acid-water (20:4.3:75.7 v/v/v). Precipitates were removed by centrifugation at 14000 *g*. Along with the peak fractions collected earlier from RPC, 50 μL of these samples were then analyzed by SEC using a mobile phase composed of the same ACN-acetic acid-water mixture. All samples were analyzed neat except for unconjugated AMB that was diluted tenfold prior to analysis.

For SEC performed using PBS-EDTA as the mobile phase, 3 μL of 2 mM AMB-PEG conjugate formulations that have been retained by 10 kDa centrifugal filters (Millipore) and 50 μL of the supernatant obtained from unconjugated AMB were analyzed. All samples dispersed in PBS-EDTA were 0.22 μm-filtered (Millipore) prior to column injection.

### MALDI-TOF Analysis

The MALDI-TOF data was acquired on a 5800 MALDI-TOF/TOF mass spectrometer (AB Sciex, Foster City, CA) in positive reflectron mode. A 0.5 μl aliquot of AMB-PEG conjugation mixture or HPLC-fractionated samples was co-spotted with 0.5 μl of 2,5-dihydroxybenzoic acid (DHB) matrix at 10 mg/mL (Waters Corporation) dissolved in 80% (v/v) methanol in water. The 4700 calibration standard, Calmix (AB Sciex), was used as an external calibrant for the MS mode. The laser intensity used to acquire the data was set at 50%.

### In Vitro Toxicity in Mammalian Cells

HEK-293 (ATCC CRL-1573) and IMR-90 (ATCC CCL-186) cells were seeded in 96-well plates at a concentration of 10^4^ cells per well in DMEM (Life Technologies, Carlsbad, CA) supplemented with 10% fetal bovine serum (Life Technologies). Quantified AMB-PEG stock solutions, unconjugated AMB suspensions and buffer solutions were serially diluted in PBS-EDTA. Diluted compounds were then added to the cells at the test concentrations. Cell viability was determined using the LIVE/DEAD® Viability/Cytotoxicity Kit (Life Technologies) after 24 hours of exposure, according to manufacturer’s instructions. Briefly, spent media was removed and 100 μl of a solution containing both calcein AM and ethidium homodimer-1(EthD-1) was added to each well to a final concentration of 2 μM and 1 μM respectively. Images of the live and dead cells within individual wells were visualized using the Acumen eX3 microplate cytometer (TTP Labtech, Cambridge, UK), at the appropriate excitation and emission wavelengths, and overlaid to form a composite image representing cell viability. Experiment was repeated twice, each time with three independently prepared AMB-PEG formulations.

### In Vitro Antifungal Activity

Susceptibility testing on fungi was carried out based on the broth microdilution method (M27-A3) recommended by the Clinical and Laboratory Standards Institute [25]. Reference strains of *Candida albicans* SC5314, *Saccharomyces cerevisiae* BY4771, *Cryptococcus neoformans* (ATCC 24067) and *Aspergillus fumigatus* (ATCC MYA-1163) were tested. Test media was filter-sterilized RPMI1640 media with L-glutamine and without sodium bicarbonate (Sigma-Aldrich), adjusted to a pH of 7.0 with 0.165M 3-(*N*-morpholino) propanesulfonic acid (MOPS) (Sigma-Aldrich), with and without supplementation with 10% fetal bovine serum (FBS) (Life Technologies).

Solutions of AMB-PEG conjugate formulations were prepared, quantified and twofold serial dilutions were performed using media in 96-well flat-bottomed plates. Buffer solutions and a suspension of unconjugated AMB were used as controls. Fungal colonies were picked from streaked agar plates and resuspended in PBS. For *A*. *fumigatus*, the resulting mixture was passed through a 35 μm cell strainer (BD Falcon, Franklin Lakes, NJ) to remove the fungal filaments from the spore suspension. Next, fungal cells or spores (in the case of *A*. *fumigatus*) were enumerated using a hemocytometer. Following dilution in test media, an initial inoculum of 10^4^ cells per well was then added to individual wells. *A*. *fumigatus and C*. *neoformans* test plates were incubated at 37°C and plates inoculated with *C*. *albicans* and *S*. *cerevisiae* were incubated at 30°C. Incubation times were 24 hours for all species tested except for *C*. *neoformans* which was incubated for 48 hours, after which the MIC_50_ determined visually using a microscope. The minimum inhibitory concentration (MIC_50_) of AMB and AMB-PEG formulations was defined as the lowest drug concentration where there is a greater than 50% reduction in fungal growth.

## Results

### Preparation of AMB-PEG Formulations

**Fig 1:**
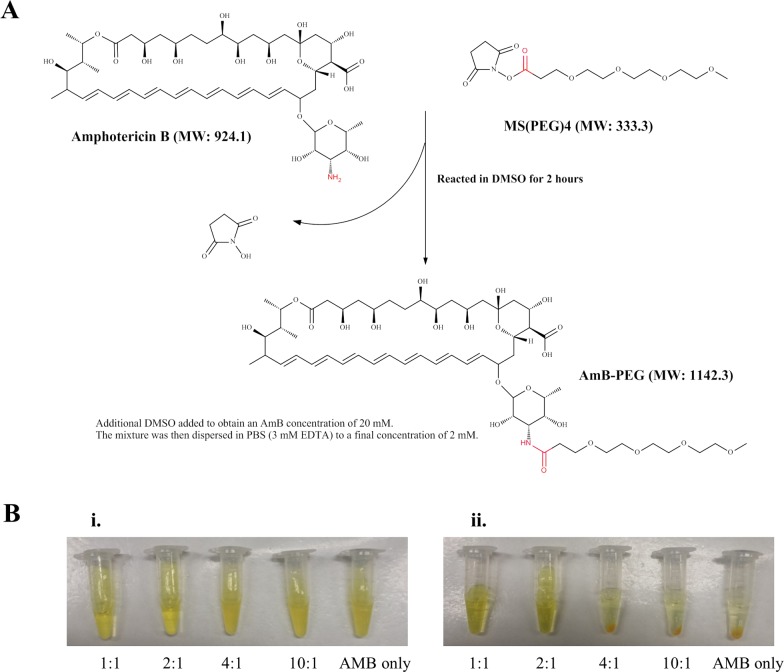
(A) AMB-PEG reaction scheme. The NHS ester on MS(PEG)_4_ reacts with the primary amine (–NH2) group on AMB at a 1:1 molar ratio, generating conjugated AMB-PEG via amide bond formation. (B) Solubility of AMB-PEG at varying AMB:PEG molar ratios. AMB-PEG mixtures and unreacted AMB were prepared in DMSO, incubated for 2 hours and then (i) dispersed to a final concentration of 2 mM in PBS-EDTA, containing 10% DMSO by volume. (ii) The mixtures were then centrifuged to facilitate the observation of insoluble precipitates. Higher molar ratios yielded a suspension of yellow particles that precipitated upon centrifugation, similar to what was observed with unconjugated AMB.

MS(PEG)_4_ consists of four ethylene glycol units functionalized with an amine-reactive N-hydroxysuccinimide (NHS) ester group at one end. The NHS ester reacts selectively and more efficiently with primary amines compared to other functional groups like carboxylic acids [26]. As AMB contains only one primary amine (-NH_2_), the NHS ester will specifically react with that primary amine (-NH_2_) functional group on AMB, forming an amide bond yielding an AMB-PEG conjugate and releasing NHS (Fig 1A). Upon dilution of the AMB-PEG reaction mixture in PBS-EDTA to a final concentration of 2 mM AMB, a homogenous yellow solution was obtained, in contrast to a suspension of yellow precipitates when the same concentration of un-reacted AMB was diluted in the same aqueous buffer (Fig 1B). To evaluate whether additional AMB can be loaded on AMB-PEG conjugates formed through the reaction with MS(PEG)_4_, AMB-PEG formulations reacted at increasing AMB:PEG molar ratios of 1:1, 2:1, 4:1 and 10:1, were diluted in PBS-EDTA (Fig 1B). Despite the two-fold loading of AMB in the 2:1 mixture, yellow solutions with no precipitates were obtained when mixtures with AMB:PEG molar ratios of 1:1 and 2:1 were resuspended in PBS-EDTA, but at higher molar ratios a yellow suspension of insoluble AMB was observed. Given the low solubility of unreacted AMB, this suggests that free AMB in the 2:1 formulation may be associated with the soluble AMB-PEG conjugate thereby increasing its aqueous solubility, and this association may be saturated at an AMB:PEG molar ratio between 2:1 and 4:1. Further testing and characterization were only carried out on the formulations with AMB:PEG molar ratios of 1:1 and 2:1, subsequently referred to as AMB-PEG 1 and AMB-PEG 2 respectively. When these formulations and the diafiltered AMB-PEG were stored at 4°C for 4 weeks, no visible precipitation was observed at concentrations of up to 4 mM, demonstrating their stability under these conditions.

### Solubility of AMB-PEG Formulations

**Fig 2:**
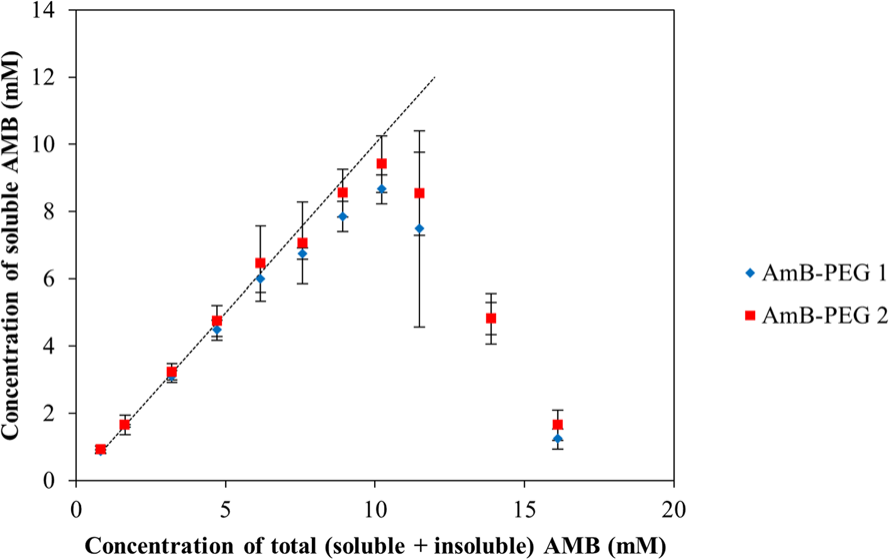
Aqueous solubility of AMB-PEG 1 and 2. Increasing amounts of AMB-PEG formulations were added to PBS-EDTA, and post-centrifugation, the concentration of soluble AMB-PEG in the supernatants quantified based on their absorbance at 365 nm. Error bars denote the standard deviation between 3 independent formulations. Dotted line represents the theoretical condition where AMB formulation is completely soluble (i.e. concentration of total AMB = concentration of soluble AMB).

To determine the maximum aqueous solubility of AMB-PEG 1 and AMB-PEG 2 that we prepared using our reaction scheme, increasing amounts of 83.3 mM AMB-PEG were added to PBS-EDTA, centrifuged at 14000 *g* to remove any insoluble AMB, and the concentration of soluble AMB-PEG quantified based on UV-visible spectrometry of suitably diluted samples (Fig 2). When small amounts of AMB-PEG were dispersed in PBS-EDTA, measured soluble AMB concentrations generally corresponded to total AMB concentrations. At high concentrations of AMB-PEG, the concentration of soluble AMB-PEG were less than the total AMB-PEG concentration and substantial precipitation was observed, suggesting an optimal concentration for the preparation of these formulations. As increasing amounts of the AMB-PEG were added to PBS-EDTA, concentrations of soluble AMB-PEG increases up to a maximum of 8.66 mM and 9.41 mM for AMB-PEG 1 and AMB-PEG 2 respectively, after which the amount of soluble AMB-PEG in the supernatant decreases significantly. Similarly prepared unconjugated AMB was practically insoluble in PBS-EDTA, in line with previously reported solubility values of crystalline of less than 0.0108 mM in water [27]. AMB-PEG conjugation thus represents a greater than 800-fold improvement in AMB solubility for both formulations.

### Particle Size of AMB-PEG Formulations

Despite having a predicted molecular weight of 1142 Da, yellow AMB-PEG was observed to be retained by a 10 kDa centrifugal filter. PEG possesses a larger specific diameter and volume compared to globular proteins of similar molecular weights and the chemical conjugation of PEG to globular proteins reduced their sieving coefficients significantly when the filter pore size was 1.5–3 times larger than their final theoretical molecular weights[28,29]. While the increase in hydrodynamic size of the AMB-PEG conjugates would be higher than what is expected from its new molecular weight, this does not fully account for its retention by the 10 kDa filter since the filter pore size is 10-times larger than the expected molecular weight of the conjugate. Hence, we postulate that AMB-PEG exists as a complex with a large hydrodynamic radius that prevents its passage through the filter.

To characterize the retained AMB, its particle size was determined using DLS. Median particle diameters were measured to be 19.8 and 31.2 nm for AMB-PEG 1 and 2 respectively (S1 Table), and inter-sample variation was minimal (< 10% of mean particle size). To better account for very large particles potentially skewing the particle size distribution within the sample, the 90% particle diameter upper limit was also recorded, and the vast majority of particles in both AMB-PEG 1 and 2 were less than 51.1 and 66.0 nm respectively. The polydispersity index (PdI) was less than 0.5 for most samples, implying that AMB-PEG conjugates are relatively homogeneous. Although AMB-PEG is smaller than AmBisome, which has a median particle diameter of 77.8 nm [30], their sizes are within the same order of magnitude, consistent with the hypothesis that the reaction of AMB-PEG complexes are formed in aqueous solutions, through the reaction of AMB and MS(PEG)_4_.

Furthermore, this may also have enabled the additional loading of free AMB beyond what is stoichiometrically expected. The median and 90% upper limit particle diameter of AMB-PEG 2 are also57.6% and 29.2% respectively larger compared to that of AMB-PEG 1, suggesting that the additional free AMB is loaded into the complex structure of AMB-PEG 2 as compared to AMB-PEG 1 This also corroborates with AMB-PEG 2 possessing similar aqueous solubility to AMB-PEG 1, even though it contains twice the amount of AMB relative to PEG.

### UV-Visible Absorbance Spectra of AMB-PEG Formulations

**Fig 3:**
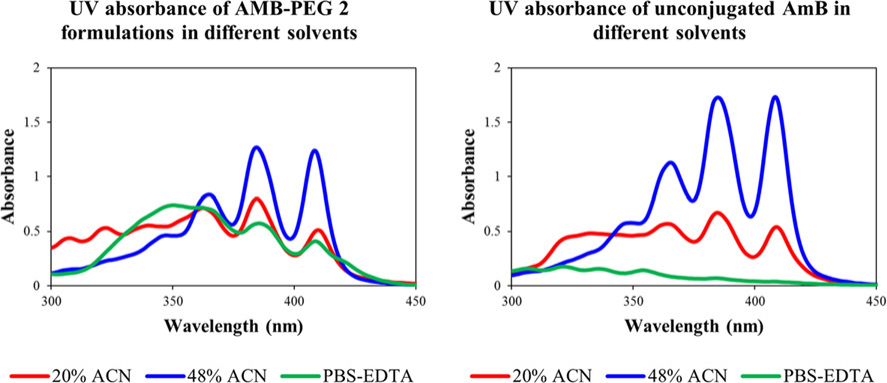
Representative absorbance spectra of AMB-PEG 2 and unconjugated AMB formulations prepared in buffers with varying hydrophobicity. 20 mM AMB-PEG 2 and unconjugated AMB formulations were prepared in DMSO and resuspended in 20% and 48% ACN buffers containing 4.3% acetic acid, as well as PBS-EDTA, to a final concentration of 2 mM AMB. As buffer hydrophobicity increases with higher ACN concentrations, the A_348_/A_409_ ratio decreases, implying that AMB-PEG is increasingly in its monomeric form. As AMB-PEG 1 and 2 have similar UV-visible absorption profiles, with identical peak height ratios in all buffers tested, data for AMB-PEG 1 is not shown. AMB-PEG formulations that have been subjected to buffer exchange to PBS-EDTA through a 10 kDa centrifugal filter have the same UV-visible absorption spectra as the initial formulation of AMB-PEG in PBS-EDTA, which contains 10% DMSO.

To further characterize these AMB-PEG formulations, the relationship between its physical properties and solvent hydrophobicity was investigated through UV-visible spectrometry. AMB possesses characteristic absorption maxima at 348, 365, 385 and 409 nm, with exact peak positions and intensities dependent on its reconstituting buffer and aggregation state, and higher A_348_/A_409_ ratios are indicative of aggregated species of AMB [31]. UV-visible absorbance spectra of AMB-PEG 1, AMB-PEG 2 and unconjugated AMB dispersed in 20% and 48% ACN buffers, as well as PBS-EDTA, were acquired (Fig 3). The absorbance profiles of the AMB-PEG formulations that have been passed through the 10 kDa centrifugal filter are identical to their unfiltered counterparts. Due to the poor aqueous solubility of unconjugated AMB, no peaks were observed when it was reconstituted in PBS-EDTA. Both AMB-PEG and unconjugated AMB possess considerably better solubility in 48% ACN compared to 20% ACN, explaining their higher peak intensities at higher ACN concentrations. Distinctive peaks corresponding to the absorbance maxima of AMB were observed in both PBS-EDTA and ACN-containing buffers. AMB-PEG 1 and 2 had similar UV-visible absorption profiles, with identical peak height ratios in all buffers tested, suggesting that the two formulations are physically similar. In PBS-EDTA, AMB-PEG formulations have a A_348_/A_409_ ratio of 1.84, which is indicative of self-aggregation [31–33]. As the polarity of the reconstituting solvent decreases with higher ACN concentrations, the A_348_/A_409_ ratio of AMB-PEG decreases, implying that proportionally more monomeric AMB-PEG is present in the mixture. This supports the hypothesis that AMB-PEG exists as a complex with a large hydrodynamic radius in aqueous solutions. Reducing the polarity of the buffer compromised the stability of these complexes, allowing the release of monomeric AMB-PEG conjugates into solution.

### HPLC Characterization of AMB-PEG Formulations

**Fig 4:**
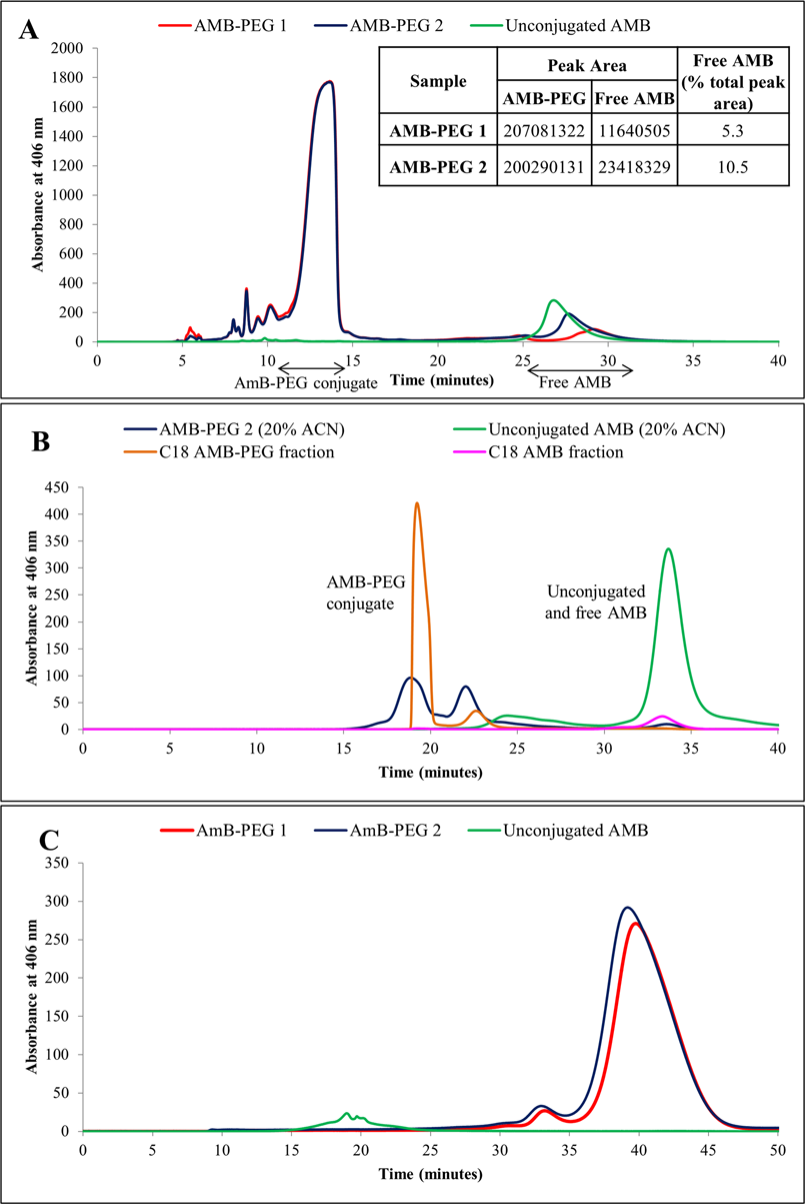
(A) Reverse phase chromatogram of AMB-PEG and unconjugated AMB, with eluted peaks detected at 406 nm. 50 μL of AMB-PEG 1 and 2, and 10 μL of unconjugated AMB dispersed in PBS-EDTA and 48% ACN respectively were injected into a C18 reverse phase column and eluted isocratically in a 48% ACN buffer at a flow rate of 0.5 ml/min for 40 minutes. Peaks were detected at 406 nm. The AMB-PEG conjugate has a shorter retention time, implying that it is more hydrophilic. From the AMB-PEG samples, AMB-PEG conjugate and free AMB (based on the retention time of unconjugated AMB) fractions were collected for further analysis via size exclusion chromatography. (B) Size exclusion chromatogram of AMB-PEG 2 and unconjugated AMB, as well as the relevant fractions collected from RPC, in a 20% ACN mobile phase. 20 mM of AMB-PEG 2 formulations and unconjugated AMB in DMSO were prepared and resuspended at 2 mM in a 20% ACN buffer. 50 μL of these samples (unconjugated AMB diluted tenfold prior to analysis) and the peak fractions collected previously from RPC were passed through a size exclusion column and eluted peaks were detected at 406 nm. Unconjugated AMB was eluted later compared to the AMB-PEG conjugate, implying that it has a smaller hydrodynamic volume compared to AMB-PEG in 20% ACN. The previously collected AMB-PEG conjugate and free AMB peak fractions had similar retention times as the AMB-PEG 2 formulation and unconjugated AMB samples respectively, thus verifying their respective peak identities. AMB-PEG 1 and 2 have identical elution profiles. (C) Size exclusion chromatogram of AMB-PEG and unconjugated AMB dispersed in PBS-EDTA. 3 μL of 2 mM AMB-PEG formulations that have been retained by 10 kDa centrifugal filters (Millipore) and 50 μL of the supernatant obtained from unconjugated AMB were analysed using a Superdex 75 size exclusion column and eluted peaks detected at 406 nm. In a PBS-EDTA mobile phase, unconjugated AMB has a shorter retention time of 20 minutes compared to AMB-PEG at 40 minutes, implying that AMB-PEG has a smaller hydrodynamic volume under these experimental conditions.

AMB-PEG formulations were then characterized by HPLC using RPC and SEC in buffers of different polarity to determine the compositions of these formulations. RPC was performed using a comparatively less polar mobile phase containing 48% ACN, which disrupts the physical interactions between free AMB and its conjugated AMB-PEG counterpart, such that the differences between the amounts of free AMB in the two AMB-PEG formulations can be discerned (Fig 4A). A peak was eluted between 25 and 31 minutes for both AMB-PEG formulations and unconjugated AMB, and was the only peak observed with the latter, thus verifying the presence of free AMB within the AMB-PEG formulations. By comparing their respective peak areas, the proportion of free AMB in AMB-PEG 2 was approximately double that of AMB-PEG 1, validating the double AMB-loading of the former and establishing that the AMB-PEG conjugate can associate with additional free AMB.

**Fig 5:**
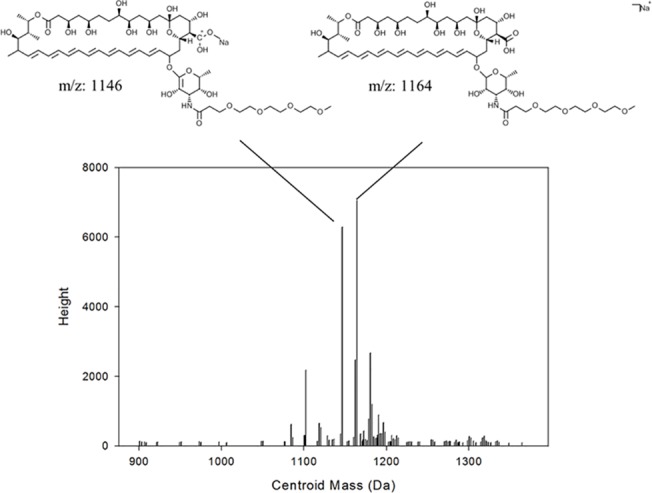
MALDI-TOF mass spectrum of the AMB-PEG fraction from RPC. The major mass peaks observed had masses corresponding to that of the AMB-PEG conjugate, thereby verifying the identity of that peak fraction. AMB-PEG mass peaks were absent from the collected unconjugated AMB fraction.

A second large peak at 12–14 minutes was observed for the AMB-PEG samples but not for unconjugated AMB (Fig 4A). This fraction was subsequently analyzed by MALDI-TOF and mass peaks corresponding to the sodium adduct of AMB-PEG conjugate were positively identified (Fig 5), confirming the identity of the peak and suggesting that the main soluble product of the reaction between AMB and MS(PEG)_4_ is the AMB-PEG conjugate. As the conjugation of AMB to hydrophilic PEG improves its aqueous solubility and reduces its interaction with the hydrophobic C18 column matrix, the retention time of conjugated AMB-PEG was shorter than that of unconjugated AMB. Peak areas for the AMB-PEG conjugate were also obtained and were comparable for both formulations, confirming that the differences in the peak corresponding to free AMB was not attributed to loading differences.

AMB-PEG 2 formulations and AMB fractions collected from RPC were analyzed through SEC using a 20% ACN mobile phase (Fig 4B) since unconjugated AMB is relatively soluble in this buffer. The AMB fraction gave a single peak eluting from 32 to 36 minutes, corresponding to the peak showed by a sample containing only unconjugated AMB. On the other hand, the AMB-PEG fraction eluted in two peaks from 19 to 24 minutes, with the peak corresponding to unconjugated AMB absent. AMB-PEG 2, prepared in the 20% ACN buffer, yielded both AMB-PEG and AMB peaks, demonstrating that it contained both species. AMB-PEG conjugates had a shorter retention time compared to unconjugated AMB, implying that their hydrodynamic volume is relatively larger in this buffer, in agreement with the observed limited solubility of AMB-PEG formulations in the same buffer (data not shown).

To determine the hydrodynamic volumes of AMB-PEG formulations and unconjugated AMB in aqueous buffers, into which they are resuspended in for efficacy and toxicity testing, SEC was carried out using PBS-EDTA as the mobile phase (Fig 4C). Unconjugated AMB was eluted earlier, with a retention time of 20 minutes compared to that of conjugated AMB-PEG at 40 minutes (Fig 4C). This suggests that unconjugated AMB has a larger hydrodynamic volume than AMB-PEG conjugates in PBS-EDTA, possibly due to its aggregation in aqueous solutions. Only a single pronounced peak was detected for both AMB-PEG 1 and 2, implying that the complexes have relatively homogeneous size distributions in PBS-EDTA, corroborating the DLS results. Both samples also possessed identical retention times and peak heights, although there was an approximately 11.4 nm size difference between AMB-PEG 1 and 2 when measured using DLS, suggesting that this difference could not be resolved using SEC. No peak corresponding to that of unconjugated AMB was observed when AMB-PEG formulations were analyzed although such peaks were present in previous HPLC analyses (Fig 4A and 4B), indicating that the additional AMB present in AMB-PEG 2 is likely to be physically associated as a complex with the conjugated AMB-PEG in aqueous PBS-EDTA. Due to its poor aqueous solubility, unconjugated AMB is likely to remain associated with the AMB-PEG conjugate, which is in turn stabilized by the hydrophilic aqueous environment provided by PBS-EDTA.

### In Vitro Toxicity in Mammalian Cells

**Fig 6:**
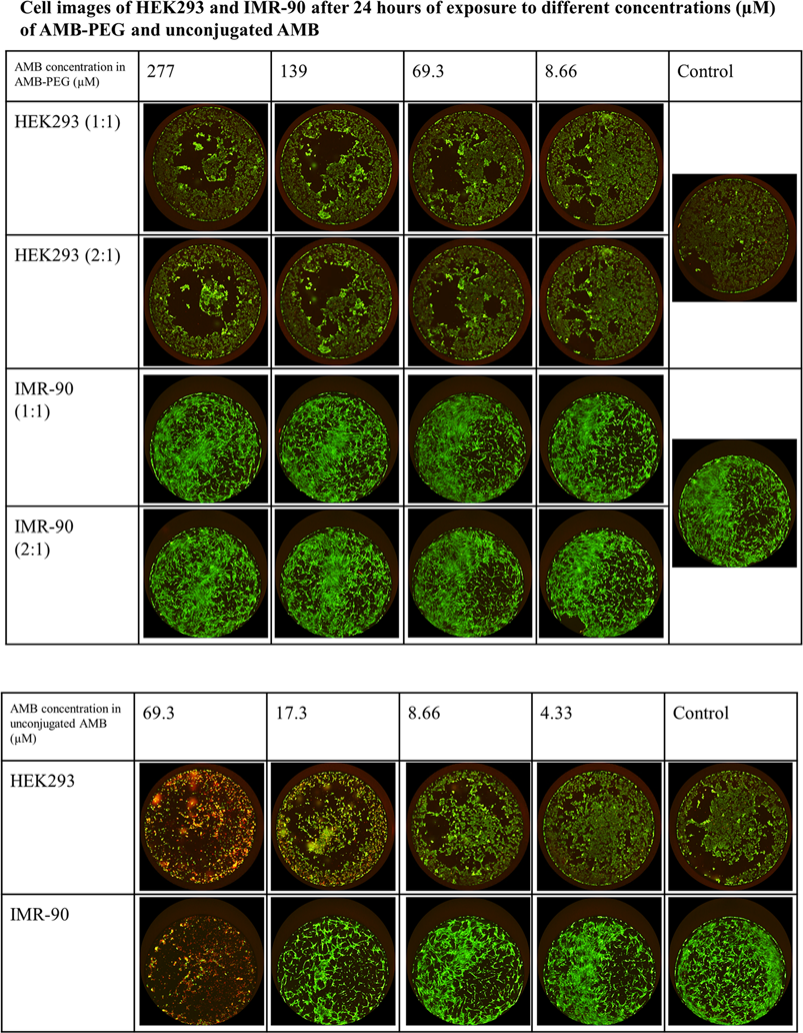
LIVE/DEAD staining of HEK293 and IMR-90 cells after exposure to AMB-PEG 1, 2 and unconjugated AMB for 24 hours. Live cells are stained green and dead cells stained red. AMB-PEG did not cause cell death at concentrations of 139 μM in HEK293 cells and 277 μM in IMR-90 cells. The molar ratio of AMB to PEG did not have any visible effect on cell toxicity. Conversely, unconjugated AMB caused extensive cell death at concentrations above 4.33 μM in both cell lines. Experiment was performed twice, each time with three independently prepared AMB-PEG formulations.

HEK-293 (a human embryonic kidney immortalized cell line) and IMR-90 (a human primary lung fibroblast cell line) cells were used to determine the *in vitro* toxicity of the AMB-PEG formulations in mammalian cells. In both cell lines tested, AMB-PEG formulations were less cytotoxic compared to the AMB suspension. Upon addition of calcein AM and EthD-1, live cells would be stained green and dead cells stained red. Images of the stained cells at varying AMB concentrations are depicted in Fig 6. At similar concentrations tested, there were fewer dead cells and more live cells in wells treated with AMB-PEG formulations compared to those with unconjugated AMB added. For example, treatment with unconjugated AMB led to extensive cell death being observed at 69.3 μM, a concentration at which there was minimal growth inhibition observed for AMB-PEG. AMB-PEG formulations did not initiate significant cell death relative to the buffer control in IMR-90 and HEK-293 cells at concentrations as high as 277 μM and 139 μM respectively. Conversely, AMB suspension caused substantial growth inhibition and cell death in both mammalian cell lines tested at concentrations greater than 4.33 μM, suggesting that AMB-PEG formulations can be utilized at 32-fold higher concentrations than unconjugated AMB for similar *in vitro* toxicity to mammalian cells.

### In Vitro Antifungal Activity

AMB-PEG formulations were tested on *C*. *albicans*, *S*. *cerevisiae*, *C*. *neoformans* and *A*. *fumigatus* to determine their antifungal activity as compared to the unconjugated AMB suspension (Table 1). Minimum inhibitory concentrations were determined in RPMI as recommended by the Clinical and Laboratory Standards Institute [25], as well as in serum-supplemented RPMI to ensure comparability with *in vitro* mammalian cells toxicity testing conditions. Fungal growth was observed to be better in serum-containing media (data not shown), which may also better mimic *in vivo* physiological conditions.

**Table 1:** MIC_50_ (μM) of various fungal species.

		*MIC*_*50*_ *(μM)*			
Media	Test agent	*A*. *fumigatus*	*C*. *albicans*	*C*. *neoformans*	*S*. *cerevisiae*
	**Incubation parameters**	(24h, 37°C)	(24h, 30°C)	(48h, 37°C)	(24h, 30°C)
**RPMI only**	**AMB suspension**	1.08	0.541	0.541	1.08
	**AMB-PEG 1**	8.66	4.33	4.33	4.33
	**AMB-PEG 2**	8.66	4.33	4.33	4.33
**RPMI + 10% FBS**	**AMB suspension**	0.541	0.271	0.271	0.541
	**AMB-PEG 1**	34.6	8.66	8.66	8.66
	**AMB-PEG 2**	17.3	4.33	4.33	4.33

Susceptibility testing was performed using AMB-PEG formulations that have been passed through a 10kDa centrifugal filter and quantified, as well as unconjugated AMB, under stated incubation conditions. AMB-PEG 2 is approximately twice as effective at inhibiting fungal growth in serum-supplemented media. Unconjugated AMB also has better antifungal activity compared to the AMB-PEG formulations, especially in serum-containing media. Results reported are from 3 independent experiments.

In RPMI, both AMB-PEG 1 and 2 had the same MIC_50_ for all fungal species tested_,_ which were 4 to 8 times higher than that of the AMB suspension (Table 1). Compared to serum-free RPMI, AMB-PEG 1 had higher MIC_50_ values for yeast cultivated in serum-supplemented media, while the MIC50 values of AMB-PEG 2 remained the same and were 8 to 32-times higher than that of the AMB suspension (Table 1). AMB-PEG 2 also possessed a two-fold stronger inhibitory effect on fungal growth in serum-supplemented RPMI as compared to AMB-PEG 1, despite their comparable toxicity to mammalian cells. Conversely, AMB suspension had an enhanced antifungal effect in serum-containing RPMI. Possible explanations for this are the additional free AMB loaded onto the AMB-PEG 2 formulation, and differential binding of the AMB formulations to serum proteins [34]. AMB-PEG formulations also inhibited the growth of *A*. *fumigatus*, a mold, to a lesser extent compared to the other three yeast species tested in serum-containing media.

Comparing the relative *in vitro* antifungal efficacy and toxicity of the AMB-PEG formulations to AMB suspension, the toxicity of AMB-PEG 2 is halved at the effective dose for growth inhibition of *C*. *albicans* and *C*. *neoformans*.

## Discussion

PEG has been previously shown to increase the aqueous solubility of many therapeutic agents including AMB [17,21,23]. In this study, AMB was reacted with MS(PEG)_4,_ yielding an AMB-PEG formulation with significantly better aqueous solubility compared to AMB (Fig 1B). The concepts of AMB liposomal formulations and that of AMB-PEG conjugation were also combined through the generation of AMB-PEG formulations containing excess AMB. These formulations were then characterized by DLS and HPLC to determine their particle size and composition in buffers of differing polarity. Their antifungal efficacies and toxicities to mammalian cells were also evaluated.

When the particle size of AMB-PEG aqueous formulations was measured using DLS, median particle diameters between 19.8 and 31.2 nm were obtained (S1 Table), suggesting that a colloidal dispersion was obtained through complex formation, whose particle size is sufficiently small to be sterile filtered through 0.22 μm filters. This hypothesis is further supported by the formulations’ relatively high A_348_/A_409_ ratio of 1.84, which is indicative of AMB self-aggregation [31–33], and their retention by 10 kDa centrifugal filters. However, when separated by SEC using a Superdex 75 column, both AMB-PEG formulations had a retention time of 20 minutes (Fig 4B), suggesting that they were significantly smaller than the smallest globular protein standard possessing a mass of 6.5 kDa. This method of size determination via the utilization of these molecular weight standards is thus possibly inaccurate as it assumes that the standard and our sample possess highly similar structures [35,36]. Since AMB-PEG and globular proteins have largely different chemical compositions and polarity, this is a possible explanation for the difference in estimated size observed during SEC. DLS calculates particle diameter via acquiring the translational diffusion coefficient of solutes with the underlying assumption that they are perfectly spherical, leading to potential sizing inaccuracies. Non-spherical solutes are liable to migrate in unexpected ways through a size exclusion column [37,38], hindering precise size determination and possibly contributing to the size disparity observed between SEC and DLS.

Comparing their retention times in SEC using different mobile phases, AMB-PEG formulations appear to have larger hydrodynamic volumes compared to unconjugated AMB in the 20% ACN mobile phase, while the inverse was observed in PBS-EDTA. Since hydrodynamic parameters are dependent on the sample’s surface structure, conformation and shape, as well as the ionic strength of the solvent [39,40], changes in buffer composition can alter the hydrodynamic volume of the solute, resulting in the observed changes in retention time. In aqueous PBS-EDTA, unconjugated AMB may form large aggregates to minimize exposure of the hydrophobic interior to the aqueous medium, while AMB-PEG forms tight micellar structures consisting of a tight hydrophobic AMB core. Increasing the concentration of an organic component (e.g. ACN) in the buffer suppresses hydrophobic and electrostatic interactions, minimizing solute interaction with the column. When 20% ACN was used, the hydrophobic tail of unconjugated AMB may be exposed allowing for increased interaction with the organic buffer and consequently, reduced aggregation and better solubility compared to aqueous dispersions of unconjugated AMB. Orientation of hydrophobic groups may differ for AMB-PEG in 20% ACN, with a greater number facing outwards compared to PBS-EDTA, with more interactions between the different complexes increasing its hydrodynamic volume while the same buffer may allow the hydrophobic AMB core of AMB-PEG to be released to potentially form larger complexes with larger hydrodynamic volumes.

Measured using DLS, AMB-PEG 2 had a 57.6% larger median particle diameter of 31.2 nm compared to AMB-PEG 1 at 19.8 nm (S1 Table), implying that additional free AMB was loaded onto the AMB-PEG 2 complex. When characterized through RPC using a 48% ACN buffer, AMB contained within the AMB-PEG complex can dissociate from the latter, and a peak representing this free AMB was observed. The area of this peak was double in AMB-PEG 2 compared to AMB-PEG 1 (Fig 4A), thus verifying the additional loading of free AMB in AMB-PEG 2. One possible explanation for this is that the hydrophobic end of AMB-PEG may have associated physically with the additionally loaded AMB, potentially as a micellar or liposomal structure, thereby enhancing its solubility beyond the binding capacity of the functionalized MS(PEG)_4_. Further studies may be performed to investigate how temperature affects the rate of dissociation of AMB from the AMB-PEG formulation, as this is an important determinant of its *in vivo* toxicity profile.

Compared to unconjugated AMB, AMB-PEG formulations are less toxic to mammalian cells (Fig 6). Coupled with its enhanced aqueous solubility, this permits higher loading doses to be administered and thus possibly better antifungal efficacy. Even though current *in vitro* data suggests that its reduction in toxicity is mitigated by its reduction in antifungal activity (Table 1), it is probable that the therapeutic index of AMB-PEG 2 is better compared to that of unconjugated AMB for the treatment of *C*. *albicans* and *C*. *neoformans*. Nevertheless, further studies are required in murine models to elucidate its potential therapeutic efficacy and toxicity *in vivo*. AMB-PEG 2 formulations exhibited a two-fold improvement in antifungal efficacy over AmB-PEG 1formulations in serum containing RPMI (Table 1), which may be attributed to the presence of additional free AMB in AMB-PEG 2, without any corresponding increase in *in vitro* toxicity to mammalian cells or compromising the aqueous solubility of the formulation. Additional loading of freeAMB to AMB-PEG complexes may therefore be a viable strategy to further improve both the solubility and therapeutic index of AMB.

## Supporting Information

S1 Table Median particle diameters and the 90% size upper limit of 10kDa-retained AMB-PEG 1 and 2 obtained from DLS analysis. (TIF)
