# Correction: Characterization of a Polyethylene Glycol-Amphotericin B Conjugate Loaded with Free AMB for Improved Antifungal Efficacy

**DOI:** 10.1371/journal.pone.0307862

**Published:** 2024-07-23

**Authors:** Tessa Rui Min Tan, Kong Meng Hoi, Peiqing Zhang, Say Kong Ng

In Fig 6, the images of HEK293 cells stated to have been treated with AMB-PEG 1:1 at 139, 69.3 and 8.66 μM, are photos of HEK23 cells treated with AMB-PEG 2:1 at their respective concentrations. Please see the correct Fig 6 here.

**Fig 6:**
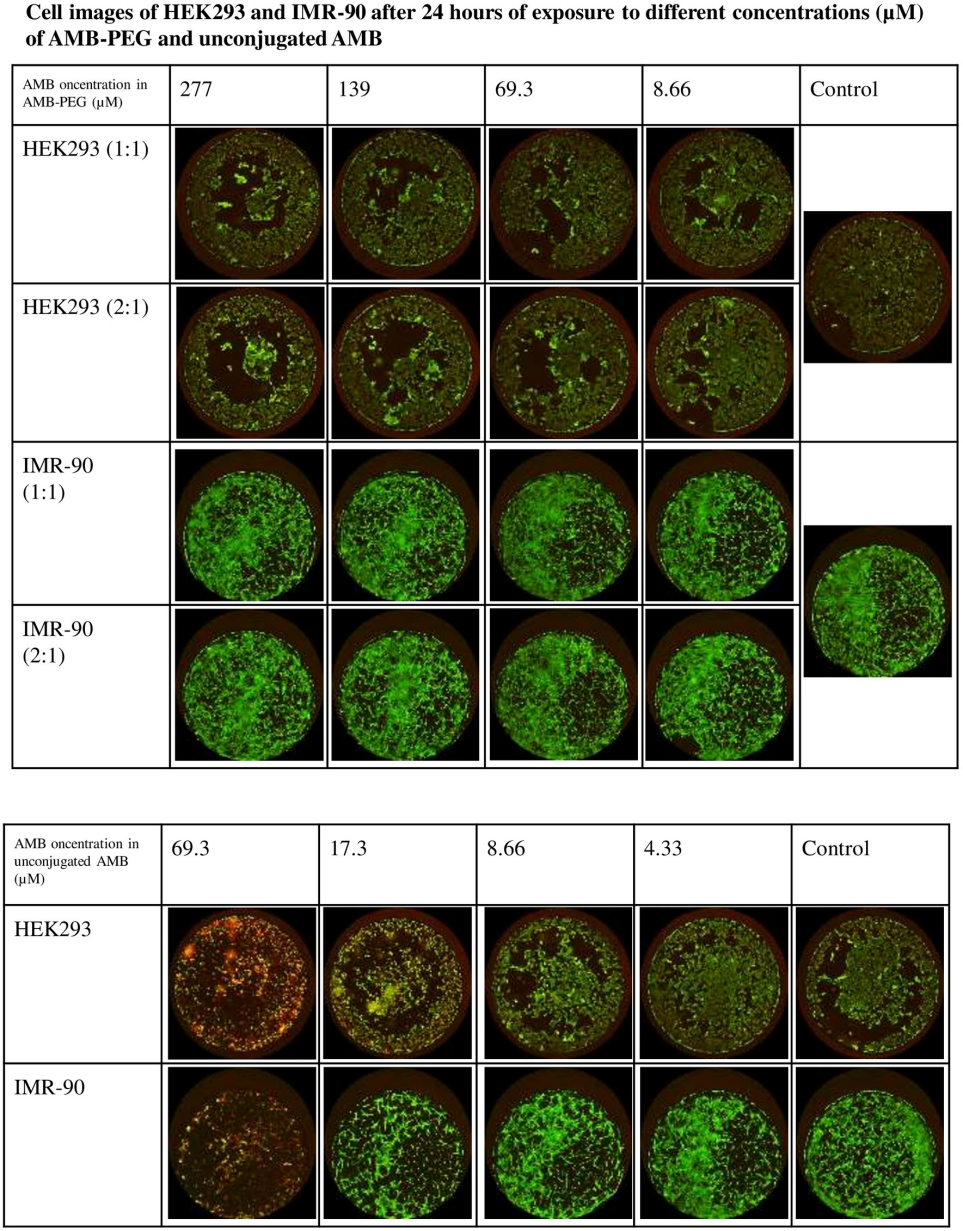
LIVE/DEAD staining of HEK293 and IMR-90 cells after exposure to AMB-PEG 1, 2 and unconjugated AMB for 24 hours. Live cells are stained green and dead cells stained red. AMB-PEG did not cause cell death at concentrations of 139 μM in HEK293 cells and 277 μM in IMR-90 cells. The molar ratio of AMB to PEG did not have any visible effect on cell toxicity. Conversely, unconjugated AMB caused extensive cell death at concentrations above 4.33 μM in both cell lines. Experiment was performed twice, each time with three independently prepared AMB-PEG formulations.

## References

[1] Tan TRM, Hoi KM, Zhang P, Ng SK (2016) Characterization of a Polyethylene Glycol-Amphotericin B Conjugate Loaded with Free AMB for Improved Antifungal Efficacy. PLoS ONE 11(3): e0152112. doi: 10.1371/journal.pone.0152112 27008086 PMC4805162

