# Supplementary figures and images for: Characterization of a Polyethylene Glycol-Amphotericin B Conjugate Loaded with Free AMB for Improved Antifungal Efficacy
Source: PLoS One. 2016 Mar 23;11(3):e0152112. doi: 10.1371/journal.pone.0152112 (PMC4805162; doi:10.1371/journal.pone.0152112)

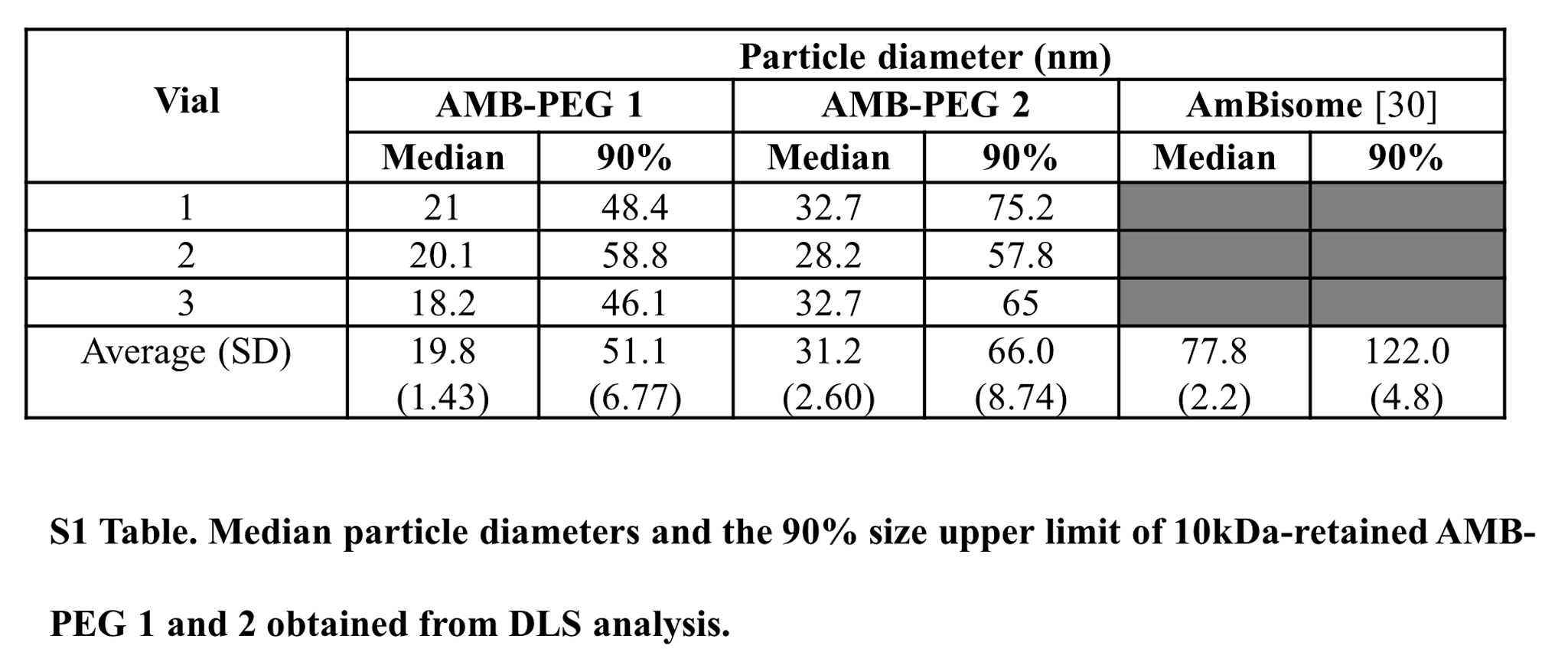

Supplement: S1 Table — (TIF) [file pone.0152112.s001.tif]
